# Incidence of atrioventricular block after isolated coronary artery bypass grafting: a systematic review and pooled-analysis

**DOI:** 10.3389/fcvm.2023.1225833

**Published:** 2023-08-01

**Authors:** Ramin Yaghoobian, Reza Hosseini Dolama, Hamidreza Soleimani, Sahar Saeidi, Mahtab Mashayekhi, Parsa Mirzayi, Ghazaal Alavi Tabatabaei, Kaveh Hosseini

**Affiliations:** ^1^ Tehran Heart Center, Cardiovascular Diseases Research Institute, Tehran University of Medical Sciences, Tehran, Iran; ^2^ Students’ Scientific Research Center (SSRC), Tehran University of Medical Sciences, Tehran, Iran; ^3^ Isfahan Cardiovascular Research Center, Cardiovascular Research Institute, Isfahan University of Medical Sciences, Isfahan, Iran; ^4^ Cardiac Primary Prevention Research Center, Cardiovascular Diseases Research Institute, Tehran University of Medical Sciences, Tehran, Iran

**Keywords:** coronary artery bypass grafting (CABG), complete heart block (CHB), atrioventricular (AV) block, temporary pacing, temporary pacemaker, permanent pacemaker (PPM), conduction defect (CD)

## Abstract

**Background and objectives:**

Atrioventricular block (AVB) is a serious complication following coronary artery bypass grafting (CABG) surgery, and its high-grade form may necessitate the implantation of a permanent pacemaker (PPM). AVB is associated with increased morbidity and mortality rates. This study aims to estimate the incidence of AVB and subsequent PPM implantation after isolated CABG surgery.

**Material and methods:**

We searched electronic databases of PubMed, Embase, and Scopus from inception to 18 November 2022. Clinical trials and observational studies reporting the incidence of post-CABG AVB or subsequent PPM implantation in adult patients were included. The total incidence for all included outcomes was calculated using the inverse variance method, and the *I*^2^ statistic was reported to evaluate the heterogeneity of studies.

**Results:**

A total of 28 studies met the inclusion criteria. Four studies [3 cohorts, 1 randomized controlled trial (RCT)] reported AVB without specifying its type; one (cohort) reported different degrees of AVB, 20 (12 cohorts, 8 RCTs) reported complete heart block (CHB) (or AVB requiring temporary pacing), and nine (8 cohorts, 1 RCT) reported the number of PPM inserted due to AVB. The pooled incidence of AVB, CHB (or AVB requiring temporary pacing), and PPM due to AVB was 1.16%, 1.73%, and 0.58%, respectively. Meta-regression analysis revealed that age, gender, diabetes, hypertension, hyperlipidemia, or smoking were not significantly associated with AVB, CHB, or PPM implantation.

**Conclusion:**

This study highlights the incidence of AVB and the need for PPM implantation following CABG surgery. The findings emphasize the importance of postoperative monitoring and surveillance to improve patient outcomes.

**Systematic Review Registration:**

https://www.crd.york.ac.uk/prospero/display_record.php?ID=CRD42022377181, identifier PROSPERO CRD42022377181.

## 1. Introduction

Coronary artery bypass grafting (CABG) surgery is a well-known therapeutic strategy for treating coronary artery disease that uses autologous arteries or veins to bypass clogged arteries. It is recommended when a severe blockage in one of the major coronary arteries cannot be relieved by percutaneous coronary intervention (1, 2). Approximately 400,000 CABG procedures are performed annually in the United States, making it one of the most performed major surgeries (1). Early cardiac complications of CABG include myocardial infarction (MI), graft occlusion, low cardiac output, vasodilatory shock, pericarditis, and arrhythmias (3). Arrhythmias encompasses bradyarrhythmias, including sinus node dysfunction and atrioventricular block (AVB) (4, 5). Surgical trauma, local edema, inflammation, and ischemia are thought to be involved in the development of AVB (6, 7). Studies have reported various incidence rates for post-CABG AVB from 0% to 13% (8, 9). Several risk factors, such as age, number of bypassed vessels, aortic cross-clamp (ACC) time, preoperative arrhythmia, and left ventricular dysfunction (LVD), have been associated with the occurrence of AVB and the need for permanent pacemaker (PPM) implantation after CABG (10–12). Pacemaker implantation is considered to be the main treatment of high-grade AVB. In most cases, AVB recovers spontaneously in the postoperative period and does not require a PPM (7). However, if high-grade AVB persists for more than seven days postoperatively, implantation of a PPM should be considered (13, 14).

Patients with high-grade AVB have a higher risk for syncope, congestive heart failure, ventricular tachycardia, asystole, or sudden cardiac death (15–17). High-grade AVB after CABG prolongs hospital stays, and as a result, it may considerably increase healthcare costs. Moreover, high-grade AVB requiring temporary pacing (TP) can increase mortality after CABG by up to 10% compared with patients without AVB (10). Besides, pacemaker implantation has several complications, such as infection, hematoma, cardiac injury, pneumothorax, thrombosis, lead malfunction, and dislodgement (18).

Despite the importance of this issue, few studies have investigated the incidence of AVB after CABG, and to our knowledge, no systematic review or meta-analysis has been performed on this subject. The present systematic review and meta-analysis aims to estimate the incidence of AVB and subsequent PPM implantation following CABG and to review associated risk factors. It helps to better understand the significance of AVB as a complication of CABG and to build the foundation for preventive measures.

## 2. Methods

### 2.1. Search strategy

This systematic review was conducted according to the Preferred Reporting Items for Systematic Review and Meta-analysis (PRISMA) statements (19). The study protocol was registered on PROSPERO (registration number CRD42022377181). Three electronic databases, PubMed, Embase, and Scopus, were comprehensively searched from inception to 18 November 2022. The search was performed by combining the keywords and medical subject heading (MeSH) terms. Customized search queries for each database are presented in Table 1.

**Table 1:** Search strategy.

Database name	Database provider	Search query	Last update	Number of studies
Pubmed	Medline	((coronary artery bypass[Title/Abstract]) OR (cabg[Title/Abstract]) OR (off-pump coronary surgery[Title/Abstract]) OR (“Coronary Artery Bypass”[Mesh]) OR (“Coronary Artery Bypass, Off-Pump”[Mesh])) AND ((atrioventricular block*) OR (atrio-ventricular block*) OR (av block*) OR (“Atrioventricular Block”[Mesh]))	18 November 2022	200
Embase	Elsevier	(“coronary artery bypass graft”/exp OR “coronary artery bypass surgery”/exp OR “off pump coronary surgery”/exp OR “coronary artery bypass”: ab,ti OR “cabg”:ab,ti OR “off-pump coronary surgery”: ab,ti) AND (“atrioventricular block”/exp OR “atrioventricular block”: ab,ti OR “atrioventricular blocks”: ab,ti OR “av block”: ab,ti OR “av blocks”: ab,ti) AND [article]/lim AND ([adult]/lim OR [middle aged]/lim OR [aged]/lim OR [very elderly]/lim) AND [humans]/lim AND [english]/lim	18 November 2022	467
Scopus	Elsevier	(TITLE-ABS-KEY (”coronary artery bypass”) OR TITLE-ABS-KEY (cabg) OR TITLE-ABS-KEY (”off-pump coronary artery”)) AND (TITLE-ABS-KEY (”atrioventricular block”) OR TITLE-ABS-KEY (”atrio-ventricular block”) OR TITLE-ABS-KEY (”av block”))	18 November 2022	573

### 2.2. Study selection and data extraction

The inclusion and exclusion criteria were as follows: randomized clinical trials and observational studies reporting the incidence of AVB or PPM implantation due to AVB after isolated CABG surgery in adult patients (18 years or older) were included. Animal studies, studies not in English, conference abstracts, review articles, research letters, or studies whose full texts were not available were excluded. After the initial search and removing duplicates, two authors screened the titles and abstracts to identify relevant studies. The full texts of potentially relevant studies were independently reviewed following inclusion and exclusion criteria, and relevant data were extracted into a pre-defined Microsoft Excel spreadsheet (Microsoft Corporation, Redmond, WA, USA). The way of consensus resolved any conflict in this step. The following variables were extracted from each study: first author, publication year, study design, sample size and the major cardiovascular risk factors (including age, sex, diabetes, hypertension, hyperlipidemia, smoking, left ventricular ejection fraction), mean follow-up time, number of AVBs, complete heart blocks (CHB) (or AVBs requiring TP) and PPMs inserted due to AVB. We also extracted the list of risk factors reported to have a statistically significant relationship with the review outcomes from the included studies.

It is worth mentioning that some studies also reported pacemaker implantations for indications other than post-CABG AVB, such as sinus bradycardia, bundle branch block, atrial fibrillation, and cardiac arrest. To maintain the focus and integrity of our analysis, we only entered data related to pacemakers that were specifically indicated for post-CABG high-grade AVB. Furthermore, while some included studies did not exclude patients with pre-existing AVB, and some did not explain whether they did, all of the studies clearly stated that they were reporting on newly developed AVBs after CABG. Due to the limited availability of studies that exclude patients with pre-existing AVB, we deemed it appropriate to include all relevant studies that reported post-CABG AVB.

### 2.3. Quality assessment

Two reviewers independently evaluated the quality of each study, and disagreements were resolved with the help of a third reviewer. The quality of observational studies was assessed following the Newcastle-Ottawa Quality Assessment Scale (20). The scale evaluates three main areas: (a) study selection, (b) comparability, (c) outcome, and the maximum score for cohort and case-control studies is 9. Study quality was considered high if the Newcastle-Ottawa Scale score was at least 7 points and categorized as good (3 or 4 points in the selection domain, 2 or 3 points in the exposure and outcome domain, and 1 or 2 points in the comparability domain). The quality of the study was otherwise considered low. We used Cochrane's Risk of Bias (RoB 2) tool (21) to assess randomized trials, which evaluates five main domains: randomization processes, deviations from intended interventions, missing outcome data, outcome measurement, and selection of reported results. We classified each domain individually as high risk, low risk, or some concerns. The overall risk of bias was considered low risk if all domains were recognized as low risk (Supplementary File S1).

### 2.4. Outcomes

The primary outcomes were the incidence of AVB, CHB (or AVB requiring TP), and PPM implantation. The secondary outcome was to determine if major cardiovascular risk factors are associated with the occurrence of primary outcomes.

AVB is a significant complication after CABG surgery that may range from transient and self-resolving to persistent and requiring medical intervention such as pacemaker implantation. High-grade AVB or AVB requiring TP increase morbidity and mortality (10, 22). Understanding the incidence of different grades of AVB is crucial in evaluating its overall burden and impact on patient outcomes. Furthermore, the need for PPM implantation is an important clinical endpoint as it reflects the long-term consequences of post-CABG AVB. In addition, we aimed to explore the association between major cardiovascular risk factors and the occurrence of AVB, high-grade AVB, and PPM implantation as our secondary outcome. This analysis seeks to identify potential risk factors that may contribute to the development of AVB and assist in risk stratification and postoperative surveillance strategies for high-risk patients.

The frequency of desired outcomes and methods used to identify them in each study are shown in Table 2.

**Table 2:** Frequency of desired outcomes and methods used to identify them.

Study	Study design	Detection methods	Sample size	AVB	CHB	PPM
Rose, 1974 (23)	Cohort	Arrhythmias were detected from bedside monitoring.	15		0	
Tchervenkov, 1983 (24)	Cohort	Using simultaneous surface ECGs and bipolar atrial electrograms.	25			
Reder, 1984 (25)	RCT	Surface ECG and bipolar electrograms recorded from temporary pacing wires	7		0	
Baerman, 1987 (26)	Cohort	ECG preoperatively, on the first and second postoperative days, then every other day through postoperative day 10, and again on the day of hospital discharge.	93		4	3
Weinstein, 1990 (27)	RCT		40		1	
Mosseri, 1991 (28)	Cohort	12-lead ECG	55		3	
Baraka, 1993 (29)	RCT	Patients were monitored with an ECG (V5). The cardiac rhythm after unclamping of the aorta was recorded continuously until sinus rhythm took place.	50		5	
Emlein, 1993 (30)	Cohort	12-lead ECG	1,614			8
Savunen, 1994 (8)	RCT	ECGs were registered preoperatively, as well as on the first, second and eighth postoperative days.	101		0	
Jegaden, 1995 (9)	RCT	An ECG was performed at the arrival of the patient in the ICU and on each of the 5 following days.	30		4	
Mustonen, 1998 (31)	Cohort	A fully computerized electrocardiography system was used to record both standard 12-lead and high-resolution signal-averaged electrocardiogram at rest.	181			5
Bhan, 1999 (32)	Cohort		62		1	0
Gol, 1999 (33)	Cohort	ECG	497		18	
Puskas, 2003 (34)	RCT	ECG	197		6	0
Onorati, 2003 (35)	Cohort	12-lead ECG was recorded preoperatively, at admission in the ICU, and then daily thereafter until hospital discharge. All patients underwent continuous ECG monitoring at least for the first 48 h postoperatively.	148		0	
Jokinen, 2004 (22)	Cohort	The immediate postoperative ECG was recorded during the first 30 min in the ICU, and thereafter ECGs were recorded every morning until the fifth postoperative day and again at the day of discharge.	180			5
Cook, 2005 (36)	Cohort	Three ECGs were examined: the preoperative ECG, the first ECG taken after admission to the cardiac surgical ICU, and the last ECG recorded before hospital discharge.	572	16	1	
Bethea, 2005 (37)	Cohort		222		4	1
Budeus, 2006 (38)	RCT	*P*-wave signal-averaged electrocardiogram. Patients received a 24 h-Holter ECG one day before surgery. A Holter ECG was performed continuously for 7 days after surgery.	110		2	
Asghar, 2009 (39)	Cohort		770		2	1
Al-Sarraf, 2010 (40)	Cohort	Postoperative arrhythmias were recorded by using telemetry for 72 h post surgery in patients who remained in sinus rhythm and longer in patients who sustained arrhythmia until they are back in sinus rhythm or until their rate is controlled for 48 consecutive hours. 12-lead ECG routinely for all patients in the first four postoperative days and prior to discharge.	2,813		5	
Rocha, 2012 (41)	Cohort		1,033	18		
Nasseri, 2014 (42)	RCT		60	1		
Pianta, 2015 (10)	Cohort	Electrocardiographic signs of AVB	3,532		288	8
Bortolussi, 2016 (43)	Cohort	Electrocardiographic signs	258	1		
Carmona, 2016 (44)	Cohort		3,097	8		
Cholley, 2017 (45)	RCT		335		22	
Todurov, 2021 (46)	Cohort	Electrocardiography (ECG in 12 standard leads), advanced electrocardiotopography (ECG in 60 leads), 24-hour (by Holter) ECG monitoring	129	4		

AVB, atrioventricular block; CHB, complete heart block; PPM, permanent pacemaker; ECG, electrocardiogram; RCT, randomized controlled trial; ICU, intensive care unit..

### 2.5. Data synthesis and statistical analysis

Baseline patient characteristics were reported in mean ± standard deviation (SD) format for continuous variables and percentage (number) for categorical variables. The total incidence for all included outcomes was calculated using data from the longest follow-up of each study and through Freeman-Tukey double arcsine transformation and back transformation in the inverse variance method (47). Between-study variance (*τ*^2^) was calculated through the restricted maximum-likelihood estimator (REML) (48). We also reported the *I*^2^ statistic to evaluate the heterogeneity of studies.

Furthermore, a meta-regression analysis was conducted to assess the relationship between desired outcomes and risk factors such as age, gender, diabetes, hypertension, hyperlipidemia, and smoking. We assessed publication bias using funnel plots and Egger's test. However, there is no universally accepted definition of what constitutes a positive result in a proportional meta-analysis. The presumption that positive results are more frequently published is not always valid for proportional studies (49, 50). R Programming language (R for Windows, version 4.2.1, Vienna, Austria), R Studio version 1.1.463 (Posit PBC, Boston, MA, United States), packages “meta” (version 5.5.0) and “metafor” (version 3.4.0), and STATA software version 16.0 (StataCorp LLC, College Station, TX, USA, version 16.0) were used for all statistical analyses.

## 3. Results

### 3.1. Search results

**Figure 1:**
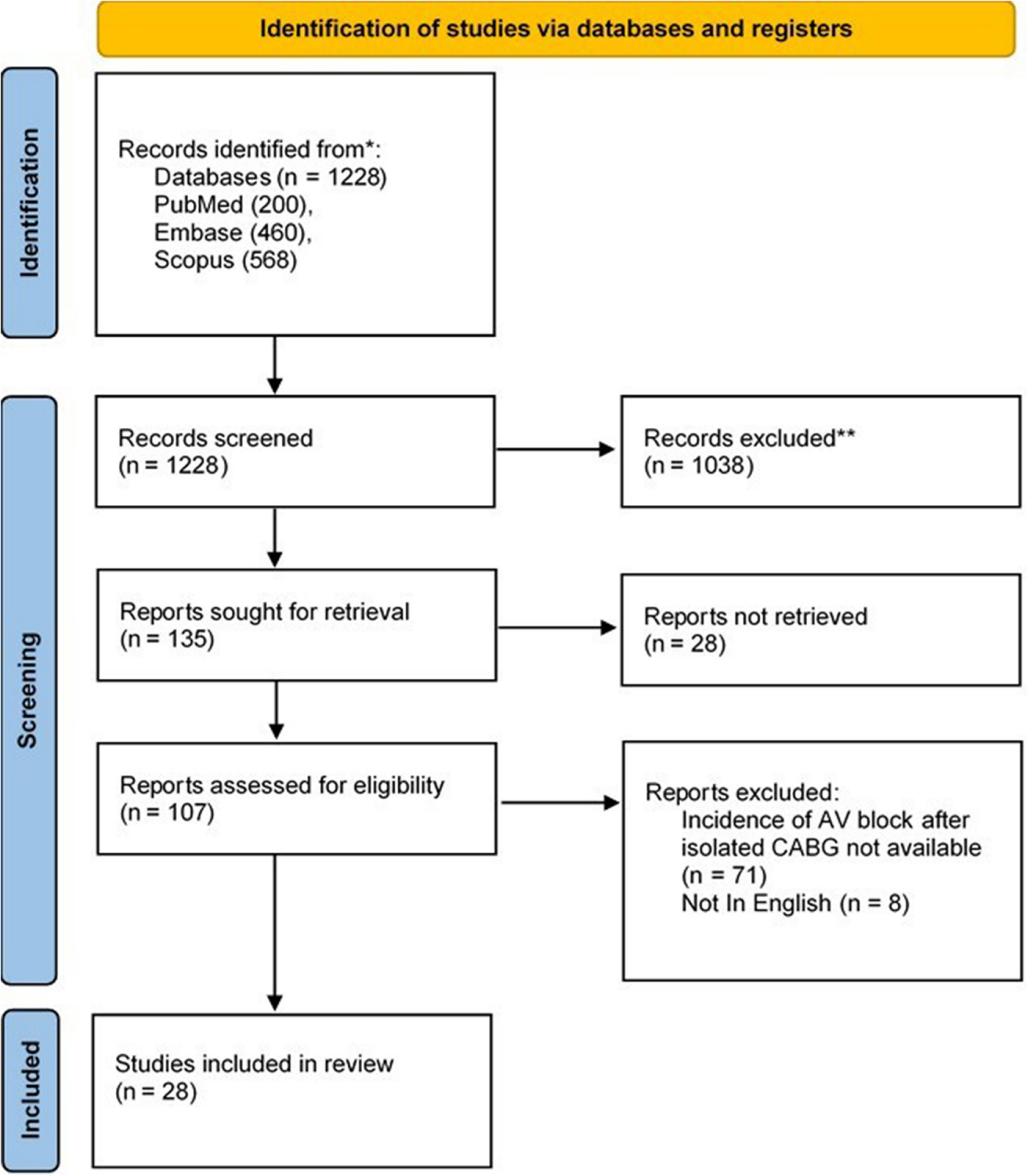
Flow diagram showing the selection of studies included in the review (PRISMA format).

The initial search of electronic databases returned 1,228 documents. After removing duplicates and initial screening, the full text of 135 articles was evaluated for inclusion and exclusion criteria. Finally, reviewers extracted the data of the thirty included articles into pre-defined spreadsheets. The PRISMA flowchart of the study is presented in Figure 1.

### 3.2. Study characteristics

The baseline demographic features and comorbidities of patients have been presented in Table 3. All observational studies that entered the review had a low risk of bias, and from nine randomized controlled trials (RCTs), three were assessed to have a high risk of bias (Table 3). The included studies reported the following postoperative outcomes: four studies reported AVB without specifying its type, one study reported different types of AVB, 20 studies reported CHB (or AVB requiring TP), and nine studies reported the number of PPM inserted due to AVB. Separate meta-analyses were performed on each of the mentioned outcomes to enhance the accuracy of the results.

**Table 3:** Baseline demographic characteristics and comorbidities.

study	Study design	Sample size	Age	Gender (female)	Diabetes	Hypertension	Hyperlipidemia	Smoking	Quality score
Rose, 1974	Prospective cohort	15							8/Low risk
Tchervenkov, 1983	Prospective cohort	25		36 (9)					8/Low risk
Reder, 1984	RCT	7		14.29 (1)					High risk
Baerman, 1987	Prospective cohort	93	61 ± 11	23.66 (22)					8/Low risk
Weinstein, 1990	RCT	40	65.18 ± 9.09	15 (6)					Low risk
Mosseri, 1991	Retrospective cohort	55	59 ± 7	12.73 (7)					8/Low risk
Baraka, 1993	RCT	50	56.79 ± 9.3						High risk
Emlein, 1993	Retrospective cohort	1,614							8/Low risk
Savunen, 1994	RCT	101		8.91 (9)					High risk
Jegaden, 1995	RCT	30	60.5 ± 9.45	23.33 (7)					Low risk
Mustonen, 1998	Prospective cohort	181							8/Low risk
Bhan, 1999	Prospective cohort	62		20.97 (13)	22.58 (14)	17.74 (11)	8.06 (5)		8/Low risk
Gol, 1999	Retrospective cohort	497		7.65 (38)	13.48 (67)	37.63 (187)	8.65 (43)	62.58 (311)	9/Low risk
Puskas, 2003	RCT	197	62.5 ± 9.81	22.84 (45)	33.5 (66)	62.94 (124)			Some concerns
Onorati, 2003	Prospective cohort	148	58.78 ± 12.18	13.51 (20)	37.16 (55)	70.27 (104)			9/Low risk
Jokinen, 2004	Retrospective cohort	180	55.7 ± 8.6	17.78 (32)	12.78 (23)	70.56 (127)			8/Low risk
Cook, 2005	Retrospective cohort	572	65 ± 10.42	23.6 (135)	28.15 (161)	55.42 (317)			8/Low risk
Bethea, 2005	Prospective cohort	222	64.91 ± 10.8	14.86 (33)	27.93 (62)	65.77 (146)	63.96 (142)	63.96 (142)	8/Low risk
Budeus, 2006	RCT	110	65.8 ± 9.57	18.18 (20)	31.82 (35)	86.36 (95)			Low risk
Asghar, 2009	Prospective cohort	770	55.88 ± 8.92	10.26 (79)	28.05 (216)	35.19 (271)	49.35 (380)		8/Low risk
Al-Sarraf, 2010	Retrospective cohort	2813	63.5 ± 9.1	19.84 (558)	19.77 (556)	57.91 (1,629)	77.11 (2,169)	41.56 (1,169)	9/Low risk
Rocha, 2012	Prospective cohort	1,033	61.98 ± 9.75	28.36 (293)	28.85 (298)	85 (878)			8/Low risk
Nasseri, 2014	RCT	60	62.3 ± 8.9	5 (3)					Low risk
Pianta, 2015	Retrospective cohort	3,532		32.25 (1,139)	31.96 (1,129)				8/Low risk
Bortolussi, 2016	Retrospective cohort	258	78.9 ± 2.82	32.56 (84)	25.58 (66)	93.02 (240)	60.08 (155)	15.12 (39)	9/Low risk
Carmona, 2016	Retrospective cohort	3,097	64.8 ± 10.18	45.01 (1,394)	42.85 (1,327)	62.16 (1,925)	57.8 (1,790)		9/Low risk
Cholley, 2017	RCT	335	68 ± 9.97	15.82 (53)					Low risk
Todurov, 2021	Prospective cohort	129	62 ± 12.5	32.56 (42)					8/Low risk

RCT, randomized controlled trial.

### 3.3. Atrioventricular block

**Figure 2:**
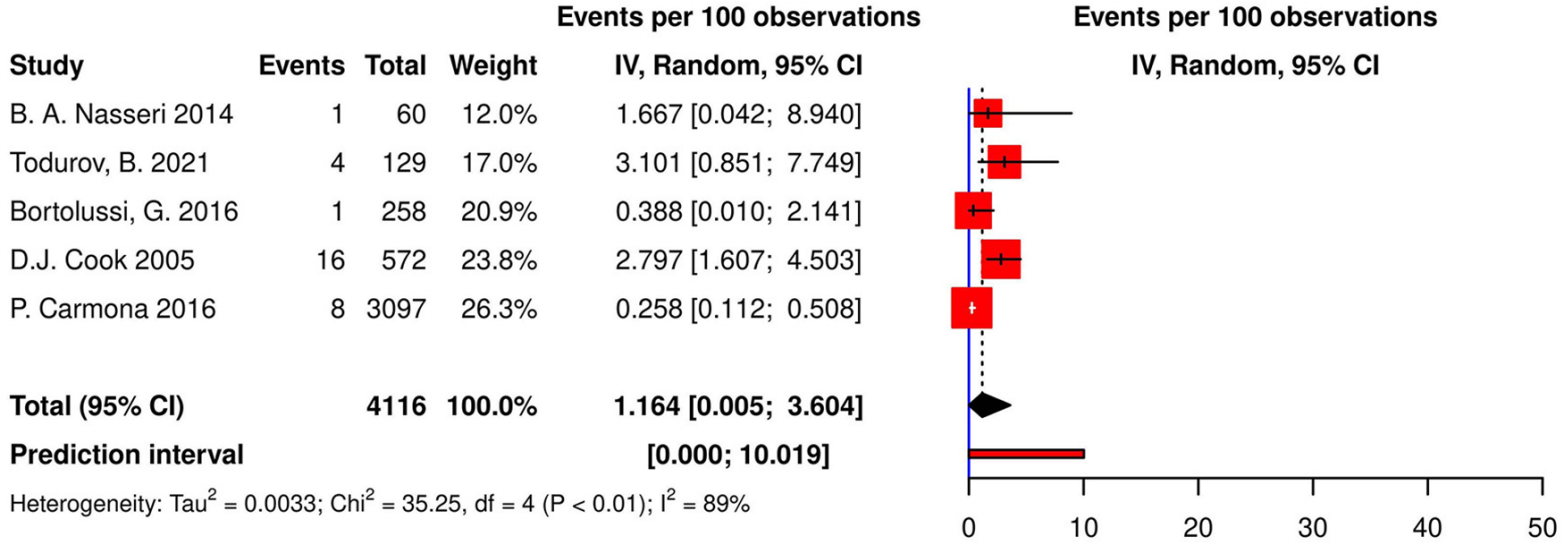
Forest plot demonstrating the incidence of AVB in patients following CABG. AVB, atrioventricular block; CABG, coronary artery bypass grafting; IV, individual value.

Five studies (4 cohorts, 1 RCT) reported AVBs following CABG. The pooled incidence of AVB was 1.16% [95% CI (0.00; 3.60), *τ*^2^ = 0.003, *I*^2^ = 88.7%; Figure 2] calculated with random effects model with a prediction interval ranging from 0% to 10.01%. Todurov et al. reported the highest incidence of post-CABG AVB (3.10%), and Carmona et al. reported the lowest incidence (0.26%) (44, 46). Furthermore, the meta-regression results indicated no significant relationship between the incidence of post-CABG AVB, age, gender, the prevalence of diabetes, and hypertension in studied populations (*p*-values of 0.425, 0.273, 0.727, and 0.611, respectively).

### 3.4. CHB, or AVB requiring TP

**Figure 3:**
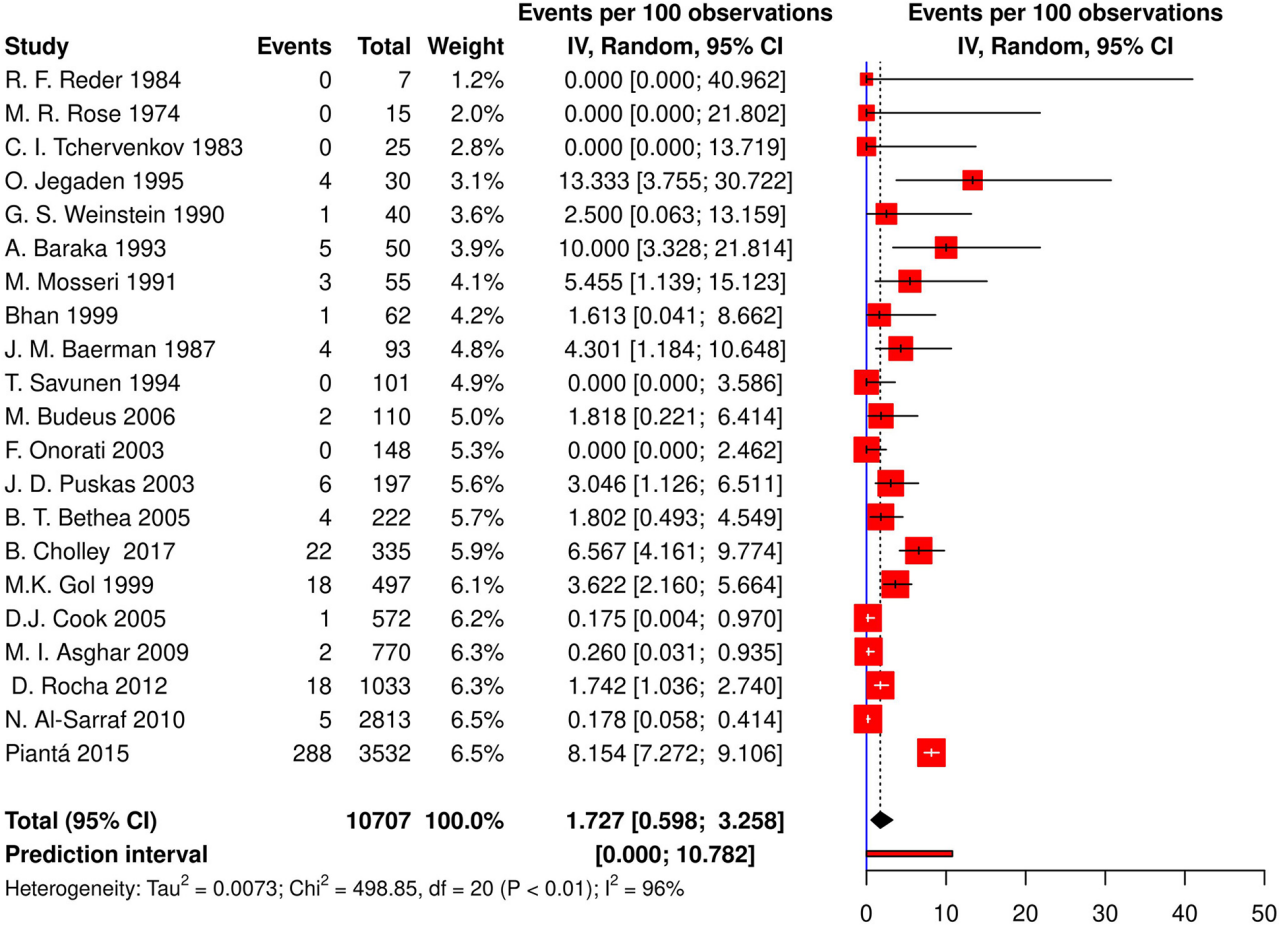
Forest plot demonstrating incidence of CHB/AVB requiring TP in patients following CABG. CHB, complete heart block; AVB, atrioventricular block; TP, temporary pacing; CABG, coronary artery bypass grafting; IV, individual value.

Twenty-one studies (13 cohorts, 8 RCTs) reported the incidence of post-CABG CHB or AVB requiring TP, resulting in an aggregate incidence of 1.73% [95% CI (0.59; 3.26), *τ*^2^ = 0.007, *I*^2^ = 96.0%; Figure 3] on random effects model. The prediction interval ranged from 0% to 10.78%. Additionally, a meta-regression of the relationship between the incidence of post-CABG CHB, or AVB requiring TP and age, gender, the prevalence of diabetes, hypertension, hyperlipidemia, and smoking was performed, which found no significant relationships (*p*-values of 0.972, 0.110, 0.366, 0.929, 0.569 and 0.619, respectively).

### 3.5. Postoperative permanent pacing due to atrioventricular block

**Figure 4:**
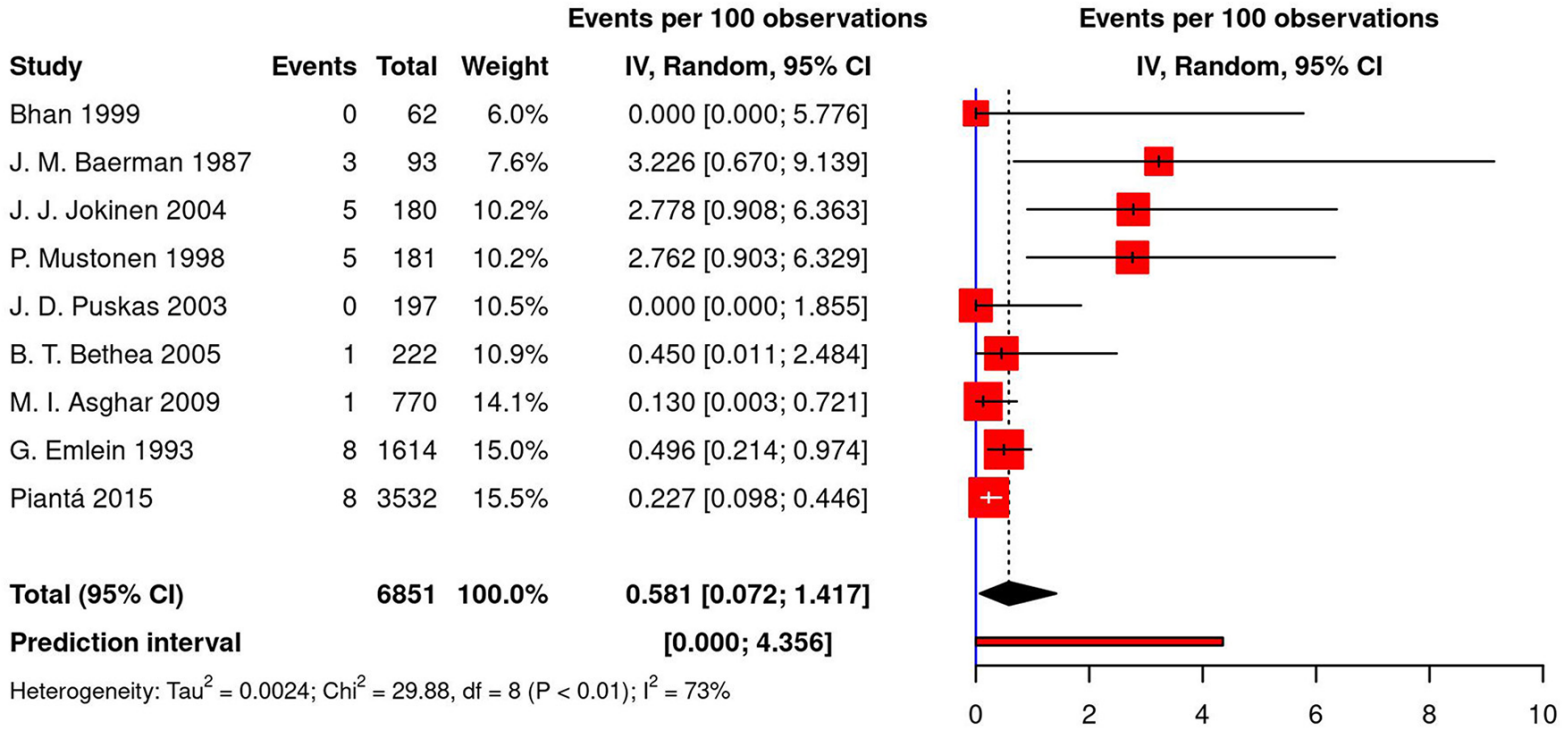
Forest plot demonstrating percentage of permanent pacing in patients following CABG. CABG, coronary artery bypass grafting; IV, individual value.

The pooled estimate for post-CABG pacing was calculated using data from nine studies (8 cohorts, 1 RCT) and equaled 0.58% [95% CI (0.07; 1.42), *τ*^2^ = 0.002, *I*^2^ = 73%; Figure 4] on the random effects model. The prediction interval for this analysis ranged from 0% to 4.35%. The highest reported percentage for permanent pacing after CABG was 3.23% (26); in two studies, no permanent pacing was required (32, 34). Moreover, in the meta-regression analysis, no significant correlation was observed between post-CABG pacing, age, gender, the prevalence of diabetes, hypertension, and hyperlipidemia (*p*-values of 0.997, 0.947, 0.784, 0.858, and 0.978, respectively).

### 3.6. Publication bias

**Figure 5:**
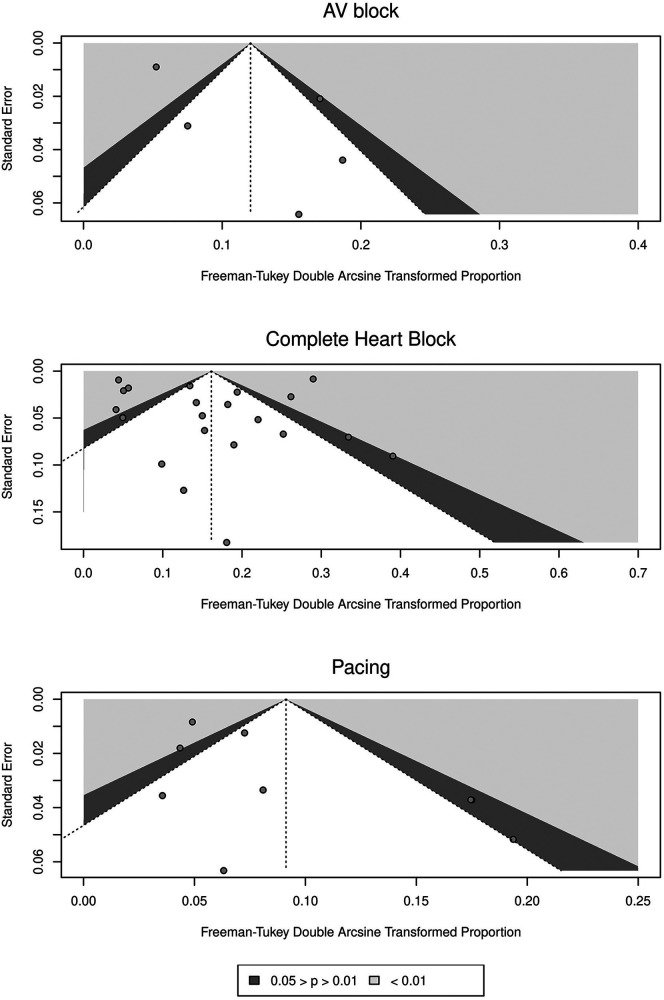
Funnel plots of standard error against freeman-tukey double arcsine transformed proportions for included studies for different outcomes.

Funnel plots were used to evaluate publication bias for all three outcomes (Figure 5). The plots for post-CABG AVB and CHB (or AVB requiring TP) were symmetrical, yet relative asymmetry was observed regarding post-CABG permanent pacing. However, Egger's test for plot asymmetry did not suggest any asymmetry for any of the outcomes. The *p*-values of Egger's test for AVB, CHB, and permanent pacing were 0.1821, 0.8425, and 0.0623, respectively.

## 4. Discussion

To our knowledge, this is the first systematic review and meta-analysis of the incidence of postoperative AVB and subsequent pacing requirements in patients undergoing isolated CABG surgery. The current meta-analysis found that after isolated CABG, the incidence of AVB in 4,116 patients was 1.16%, the incidence of CHB or AVB requiring pacemaker implantation in 10,707 patients was 1.73%, and the percentage of PPM implantation due to advanced AVB in 6,851 patients was 0.58%. Based on these results, 0.33 or one-third of patients with CHB or AVB requiring TP will need PPM implantation. In the meta-regression analysis, age, gender, diabetes, hypertension, hyperlipidemia, or smoking had no significant association with AVB, CHB, or PPM implantation. However, these variables were reported for study populations, and we were not able to access the individual data required for a more robust analysis. In this review, we considered studies that reported AVB without further explanation as high-grade AVB due to the similarity between their incidence rates and those that reported high-grade AVB requiring TP.

The results of this study show that the incidence of high-grade AVB and the subsequent need for PPM implantation is a serious complication after CABG surgery. Therefore, it is crucial to take preventive measures, especially in high-risk patients. For this purpose, the risk factors should be further investigated, and then clinical guidelines should be designed to identify high-risk patients.

### 4.1. Heterogeneity of studies

We observed a high variability between studies, especially in those reporting CHB. The overall heterogeneity between 21 studies that reported CHB or AVB requiring TP was high, with an *I*^2^ of 96.0%. The overall heterogeneity for AVB and PPM implantation was lower (*I*^2^ 88.7% and 73.2%, respectively). Since the diagnosis method was serial electrocardiograms in most studies, it does not seem to be the source of variability. Instead, two outlier studies in the CHB group (9, 29) and three outlier studies in the PPM group (22, 26, 31) with relatively small sample sizes and wide confidence intervals could be considered the primary source of heterogeneity.

In a study by Baraka et al., it was found that of 19 patients (38%) who needed pacing after releasing the ACC, only five (10%) required it upon discharge from the operating room (29). Accordingly, there is a significant difference between the number of intraoperative and postoperative AVB. In other words, many intraoperative AVBs resolve before discharge from the operating room. In confirmation of this assertion, three excluded studies reported the incidence of CHB after releasing the ACC, 16% (51), 24% (11), and 30% (12). These numbers are much higher than those reported in our included studies. In one of these studies, CHB after the release of ACC was referred to as postoperative CHB (51). Thus, there is a possibility that the studies we included also misreported postoperative AVB. In this case, this may account for some of the observed heterogeneity.

### 4.2. Risk factors

Literature has reported many risk factors associated with post-CABG AVB and the subsequent need for pacing. Here we review the risk factors in the studies we included in our meta-analysis. These consisted of old age, gender, LVD, perioperative MI, number of vessels bypassed, longer ACC time, perioperative use of an intra-aortic balloon, preoperative arrhythmia, and the type of cardioplegia (Table 4).

**Table 4:** Risk factors associated with post-CABG AVB, conduction defect or arrythemia, and pacing .

	AVB	Conduction defect or arrythemia	Pacing
Age	Pianta, Baraka (10), Rocha, Caspi (50)	Cook, Emlein, Al-sarraf	Bethea, Asghar, Puskas
Gender	Pianta (female)	Al-sarraf (male)	Puskas (female)
LVD	Pianta, Caspi	Al-sarraf, Jokinen	
Perioperative MI	Pianta, Caspi	Jokinen	
ACC time	Baraka (10)	Baerman	
More vessels	Baraka (10)	Baerman, Cook	
IAB	Pianta	Cook	
Preoperative arrhythmia	Pianta (AF)	Emlein (LBBB)	Bethea, Asghar (BBB)
Type of cardioplegia	Gozal, Jegaden	Cook	

AVB, atrioventricular block; RCT, randomized controlled trial; AF, atrial fibrillation; LBBB, left bundle branch block; BBB, bundle branch block.

One of the most frequently mentioned risk factors was age. In four studies, old age was associated with a higher incidence of AVB after CABG or after releasing the ACC. Four studies had a significant association between LVD and post-CABG AVB or pacing. In a large cohort study, Pianta et al. found that perioperative MI increased post-CABG AVB requiring TP, but the same was not true for patients with previous MI. In the same study, post-CABG AVB was more frequent in patients with preoperative atrial fibrillation (10). In other studies by Emlein et al., Bethea et al., and Asghar et al. preoperative arrhythmia, especially bundle branch block, was associated with an increased risk of post-CABG bradyarrhythmias or pacing (30, 37, 39). In a study by Baraka et al., longer ACC time and more grafted vessels were associated with the incidence of AVB after releasing the ACC (11). In one study, using an intra-aortic balloon was related to the incidence of post-CABG AVB (10). Few included studies suggested a relationship between gender and AVB or post-CABG pacing. Pianta et al. suggested that post-CABG high-grade AVB appeared more frequently in females (10). Also, females were more likely to experience post-CABG pacing in a study by Puskas et al. (34).

The type of cardioplegia can be a risk factor since different types of cardioplegia methods have been related to increased postoperative conduction disorders. A study by Cook et al. suggested that crystalloid cardioplegia increased post-CABG conduction defects compared with blood cardioplegia (36). Gozal et al. indicated that hypothermic blood cardioplegia was associated with more CHB than normothermic cardioplegia (12). In another study by Jegaden et al., antegrade cardioplegia caused more AVB requiring pacing than combined antegrade-retrograde cardioplegia (9). The other factor that has been proposed is the type of CABG surgery. Carmona et al. showed that the incidence of AVB following on-pump and off-pump CABG was not significantly different (44).

The other factor that should be further investigated is the effect of medications. Pianta et al. found that the incidence of high-grade AVB after CABG was similar between patients using beta-blockers and those without (10). In a cohort study on the prognosis of permanent conduction defects related to CABG by Jokinen et al., calcium channel blockers and beta-blockers were not associated with increased conduction defects (22). In a single-center retrospective study, Cook et al. compared two cohorts of patients who underwent CABG surgery, one in 1991 and the other in 2001, regarding conduction disorders. In 2001, compared to 1991, more patients were using beta-blockers; the opposite was true for calcium channel blockers. In the 2001 cohort, the incidence of post-CABG conduction defects decreased, but the incidence of different degrees of AVB did not differ (36). In other studies, Puskas et al., Bethea et al., and Asghar et al. found that preoperative beta-blocker use did not increase the risk of pacemaker implantation after CABG (34, 37, 39).

Because only one study (10) specifically investigated the potential association of medications with the incidence of post-CABG AVB or pacemaker implantation due to AVB, we could not analyze this association. Table 5 includes an outline of the studies that provided information about the medications used by patients.

**Table 5:** Studies that provided information about the medications used by patients.

Study	Objective	Medications
Pianta	To investigate the association between perioperative factors and the emergence of AVB in the postoperative period of CABG.	BBs, statins, digoxin, other antiarrhythmics did not prove to be independent risk variables for AVB
Cook	To identify the incidence of new CDs in the perioperative period and compare it between two cohorts of patients one in 1991 and the other in 2001.	In 1991 more BBs, in 2001 more CCBs AVB between 1991 and 2001 not different
Bethea	To identify patient characteristics predicting the need for pacing after CABG surgery with the potential to limit their utilization.	BBs and antiarrhythmics were not associated with increased pacing in multivariate analysis
Asghar	To provide data identifying patient characteristics that could predict the need for pacing after routine CABG with the potential to reduce its indiscriminate use.	BBs were not associated with increased pacing
Puskas	To reevaluate the routine use of pacing wires and to attempt to identify a subpopulation(s) of CABG patients for whom pacing wires are appropriate.	BBs were not associated with increased pacing
Jokinen	To determine the long-term prognostic significance of new permanent CDs related to CABG	CCBs and BBs were not associated with increased CDs
Mustonen	To evaluate the long-term effects of CDs on physical performance and ability to work, left ventricular function, and myocardial late potentials	CCBs, BBs, Digoxin, Diuretics were not different between groups with and without CDs
Cholley	To assess the ability of preoperative levosimendan to prevent postoperative low cardiac output syndrome.	Between Levosimendan and Placebo: BBs, Statins, Anticoagulant therapy, Antiplatelet therapy not different AVB not different
Budeus	To assess the ability of preoperative amiodarone to reduce the incidence of atrial fibrillation in high-risk patients undergoing CABG.	Between Amiodarone and Placebo: BBs, Statins, ACE inhibitors, Verapamil, Digitalis not different AVB not different
Weinstein	To assess the protective effect of pretreatment with verapamil on myocardial injury.	Between Verapamil and Control Preoperative CCBs not different AVB not different
Baraka	To assess the efficacy of lidocaine addition to crystalloid cardioplegic solution for prevention of reperfusion ventricular fibrillation in patients undergoing CABG as compared with patients undergoing mitral or aortic valve replacement.	Between Lidocaine and Control CCBs and BBs not different AVB not different

AVB, atrioventricular block; CABG, coronary artery bypass grafting; BB, beta-blocker; CCB, calcium channel blocker; CD, conduction defect; ACE inhibitor, angiotensin-converting enzyme inhibitor.

### 4.3. Prognosis

Preventing post-CABG AVB and its related complications is crucial to avoid adverse consequences. Several studies suggest the incidence of AVB after CABG surgery is associated with increased mortality. Pianta et al. indicated AVB requiring TP prolonged hospital stay and increased mortality after CABG (10). In a study by Jokinen et al. that investigated the long-term prognosis of permanent conduction defects after CABG, CHB showed a significant association with increased mortality (22). In another study by Caspi et al., AVB after releasing the ACC was associated with postoperative low cardiac output and higher mortality rates (51).

### 4.4. Limitations

There are several restrictions on how broadly applicable our findings can be. First, we only searched three online databases and included full-text English journal articles, which may have resulted in selection bias. Second, five studies reported post-CABG AVB without further explanation of its type, which limited analysis of the incidence of different types of AVB. However, we analyzed them separately to prevent the pooling of heterogeneous data. Third, studies did not separately report characteristics in patients with and without AVB, except in one (10). Consequently, we could not accurately analyze their potential associations with post-CABG AVB. Fourth, since almost all studies did not provide a precise definition of postoperative AVB, intraoperative AVB may also have been reported.

### 4.5. Clinical implications and recommendations

Our study provides important insights into the incidence of post-CABG AVB. The pooled prevalence rates calculated for the incidence of CHB (or AVB requiring TP) and PPM implantation can serve as reference points for clinicians. These incidence rates highlight the need for increased postoperative surveillance and monitoring to reduce the risk of high-grade AVB sequelae. The identification of high-risk patients should be prioritized. Risk factors such as age, preoperative arrhythmia, LVD, perioperative MI, and the use of certain medications should be considered when assessing the patients.

As for future research, we recommend conducting large-scale prospective cohort studies investigating the incidence of post-CABG AVB, its risk factors, and its long-term prognosis. These studies would provide a more comprehensive understanding of the multiple factors influencing post-CABG AVB and facilitate the development of risk prediction models. Moreover, including other patient-centered outcomes, such as quality of life measures and healthcare resource utilization, would contribute to a more holistic assessment of the impact of post-CABG AVB. The STROBE guidelines should be used for complete and transparent reporting.

One of the most significant limitations of this review was the nonuniform definitions and reporting across different studies. It underscores the importance of a uniform definition and diagnostic criteria for post-CABG AVB, which should be concise, clear, and clinically relevant. This consistency will allow for more accurate comparisons and assessment of the incidence and outcomes of post-CABG AVB across different studies. The following are our recommendations in this regard:

1. Determine whether the study focuses on developing new-onset AVB or worsening pre-existing AVB.
2. Identify the type of AVB of interest (e.g., first-degree, second-degree, third-degree).
3. Determine the time frame for defining post-CABG AVB (e.g., starting upon discharge from the operating room until one month after surgery).
4. Define the criteria for determining the presence of AVB. It could include identifying new electrocardiographic findings consistent with the type of AVB of interest.
5. Provide clear exclusion criteria. It could include:
  • Pre-existing AVB.
  • pre-existing pacemakers or implantable cardioverter-defibrillators.
  • Previous cardiac surgeries.
  • Severe comorbidities that may affect the study outcomes, such as mortality.
  • Use of medication that can exacerbate AVB.
  • Inadequate medical records.

## 5. Conclusion

The current study was designed to determine the overall incidence of AVB, CHB, and subsequent PPM implantation following the CABG procedure and identify its associated risk factors. We calculated a pooled prevalence of 1.16% for postoperative AVB incidence, 1.73% for CHB or AVB requiring TP, and 0.58% for PPM implantation. These findings underscore the significance of AVB as a serious complication of CABG, emphasizing the need for postoperative monitoring and surveillance to ensure satisfactory patient outcomes. As for further research, we recommend conducting large-scale prospective cohorts with primary outcomes focusing on the incidence, risk factors, and prognosis of post-CABG AVB to yield more precise and comprehensive results. Using a uniform definition and diagnostic criteria for post-CABG AVB and adhering to the reporting standards will allow for more accurate comparisons and assessment of the incidence and outcomes of post-CABG AVB across different studies.

## Data Availability

The original contributions presented in the study are included in the article/**Supplementary Material**, further inquiries can be directed to the corresponding author.
