# Supplementary material for: Incidence of atrioventricular block after isolated coronary artery bypass grafting: a systematic review and pooled-analysis
Source: Front Cardiovasc Med. 2023 Aug 1;10:1225833. doi: 10.3389/fcvm.2023.1225833 (PMC10427724; doi:10.3389/fcvm.2023.1225833)
Supplement: Supplementary file 1 [file Table1.docx]

Table S1. Newcastle–Ottawa scale (NOS)

| Selection | | | | | Comparability | | Outcome | | |  |
| --- | --- | --- | --- | --- | --- | --- | --- | --- | --- | --- |
| Study | Representativeness of the exposed cohort | Selection of the non exposed cohort | Ascertainment of exposure | Demonstration that outcome of interest was not present at start of study | Main factor | Additional  factor | Assessment of outcome | Follow-Up Long Enough for Outcome to Occur | Adequacy of Follow-Up | Total quality  score |
| G. Bortolussi | 1 | 1 | 1 | 1 | 1 | 1 | 1 | 1 | 1 | 9 |
| B. Todurov | 1 | 0 | 1 | 1 | 1 | 1 | 1 | 1 | 1 | 8 |
| R. M. Piantá | 1 | 0 | 1 | 1 | 1 | 1 | 1 | 1 | 1 | 8 |
| P. Carmona | 1 | 1 | 1 | 1 | 1 | 1 | 1 | 1 | 1 | 9 |
| J. M. Baerman | 1 | 0 | 1 | 1 | 1 | 1 | 1 | 1 | 1 | 8 |
| D. J. Cook | 1 | 0 | 1 | 1 | 1 | 1 | 1 | 1 | 1 | 8 |
| M.K. Gol | 1 | 1 | 1 | 1 | 1 | 1 | 1 | 1 | 1 | 9 |
| M. Mosseri | 1 | 0 | 1 | 1 | 1 | 1 | 1 | 1 | 1 | 8 |
| F. Onorati | 1 | 1 | 1 | 1 | 1 | 1 | 1 | 1 | 1 | 9 |
| D. Rocha | 1 | 0 | 1 | 1 | 1 | 1 | 1 | 1 | 1 | 8 |
| G. Emlein | 1 | 0 | 1 | 1 | 1 | 1 | 1 | 1 | 1 | 8 |
| J. J. Jokinen | 1 | 0 | 1 | 1 | 1 | 1 | 1 | 1 | 1 | 8 |
| N. Al-Sarraf | 1 | 1 | 1 | 1 | 1 | 1 | 1 | 1 | 1 | 9 |
| P. Mustonen | 1 | 0 | 1 | 1 | 1 | 1 | 1 | 1 | 1 | 8 |
| C. I. Tchervenkov | 1 | 0 | 1 | 1 | 1 | 1 | 1 | 1 | 1 | 8 |
| M. R. Rose | 1 | 0 | 1 | 1 | 1 | 1 | 1 | 1 | 1 | 8 |
| B. T. Bethea | 1 | 0 | 1 | 1 | 1 | 1 | 1 | 1 | 1 | 8 |
| A. Bhan | 1 | 0 | 1 | 1 | 1 | 1 | 1 | 1 | 1 | 8 |
| M. I. Asghar | 1 | 0 | 1 | 1 | 1 | 1 | 1 | 1 | 1 | 8 |

Table S2. Revised Cochrane risk-of-bias tool for randomized trials (RoB 2)

| Study | Domain 1 | Domain 2 | Domain 3 | Domain 4 | Domain 5 | Overall |
| --- | --- | --- | --- | --- | --- | --- |
| B. A. Nasseri | Low | Low | Low | Low | Low | Low risk |
| B. Cholley | Low | Low | Low | Low | Low | Low risk |
| M. Budeus | Low | Low | Low | Low | Low | Low risk |
| G. S. Weinstein | Low | Low | Low | Low | Low | Low risk |
| O. Jegaden | Low | Low | Low | Low | Low | Low risk |
| T. Savunen | Some concern | High | Low | Low | Low | High risk |
| A. Baraka | Low | High | Low | Low | Some concern | High risk |
| R. F. Reder | Some concerns | High | Low | Low | Low | High risk |
| J. D. Puskas | Some concerns | Low | Low | Low | Low | Some concerns |
